# Successful treatment of *Candida parapsilosis* and *Pseudomonas aeruginosa* infection using medical and surgical management in an injecting drug user with mitral and aortic valve endocarditis: a case report

**DOI:** 10.1186/1752-1947-3-6598

**Published:** 2009-05-08

**Authors:** Hanady Daas, Fadi Abuhmaid, Marcus Zervos

**Affiliations:** 1 Department of Internal Medicine, Henry Ford Hospital, 2799 W Grand Blvd, Detroit, MI 48202, USA; 2 Department of Infectious Disease, Henry Ford Hospital, 2799 W Grand Blvd, Detroit, MI 48202, USA; 3 Wayne State University, Detroit, MI, USA

## Abstract

**Introduction:**

Polymicrobial endocarditis is a well-recognized problem in intravenous drug users and it accounts for 1 to 3% of endocarditis cases overall and up to 9% in other series. The most common combinations of organisms include *Staphylococcus aureus* and *Streptococcus pneumoniae* followed by *Staphylococcus aureus* and *Pseudomonas aeruginosa*. *Candida parapsilosis* endocarditis carries a mortality rate of 45%, and each infection with *Candida* or *Pseudomonas* endocarditis per se carries a very high mortality rate approaching 85% and 80%, respectively. The combination of *P. aeruginosa* and *C. parapsilosis* has never been encountered and there have been no earlier reports of the combination of *C. parapsilosis* and *P. aeruginosa* in adult intravenous drug users as a cause of endocarditis.

**Case presentation:**

We present a 49-year-old man with bivalvular endocarditis with *P. aeruginosa* and *C. parapsilosis*. He had a prior bivalvular replacement in 2005 that became infected with the above microorganisms and he was treated with intravenous antibiotics. Because of ongoing intravenous drug use, a second valve replacement was denied. A few days later, the patient presented with septic shock secondary to *P. aeruginosa* and *C. parapsilosis* recurrent endocarditis. The infection was cured with a second bivalvular replacement and extended therapy with antibiotics and antifungals.

**Conclusion:**

This is the first time a patient has presented with *P. aeruginosa* and *C. parapsilosis endocarditis*. Relapsing polymicrobial endocarditis can be cured with medical and surgical therapy.

## Introduction

Few data exist on infective endocarditis in intravenous drug use (IVDU) patients. A new pattern of infective endocarditis in IVDU is emerging, characterized by more frequent left heart involvement, a severe clinical course, and a need for surgery in the active phase [1]. The standard of care has included perioperative antifungal, radical debridement of infected tissue, reconstruction using biological tissue when possible, and prolonged oral suppressive antifungal therapy. Medical treatment of fungal endocarditis on prosthetic valves can be successful in selected cases. Left-sided endocarditis compared to right, and polymicrobial compared to single organism, are well known risk factors for an increase in morbidity and mortality in intravenous drug users with infective endocarditis [2].

## Case presentation

A 49-year-old man with a history of intravenous drug abuse, mainly heroin, presented to the emergency department in Henry Ford Hospital where he complained of chills and diarrhea. The diarrhea was watery and continuous of 1-day duration accompanied by chills. He denied shortness of breath or chest pain.

Our patient was hospitalized in another facility 14 days before presenting to the emergency department of our hospital, and was found to have *Candida parapsilosis* and *Pseudomonas aeruginosa* endocarditis. A 2D echocardiogram was done there and showed vegetation of the prosthetic mitral valve, with normal aortic prosthesis. He was discharged with a peripherally inserted catheter (PIC) line and on intravenous antibiotics namely, tobramycin, cefepime and fluconazole. No valve replacement was performed at that time due to ongoing injection drug use. He was only out for 1 day before presenting to our emergency department.

His past medical history was significant for aortic and mitral valve replacement 1 year before this hospitalization for endocarditis. He also had stage 3 chronic kidney disease.

At initial presentation, he was found to be febrile at 100.3°F and hypotensive at a blood pressure of 85/50. His physical examination revealed a 3/6 systolic murmur in the aortic area with radiation to the precordium, and a 2/6 diastolic murmur over the mitral area without radiation.

His initial laboratory investigations showed white blood cell count of 19.2 k/μL with 93% neutrophils, hemoglobin of 9.6 g/dL, blood urea nitrogen (BUN) 26 mg/dL, creatinine 2.2 mg/dL, serum bicarbonate 16 mmol/L, chloride 112 mmol/L, sodium 136 mmol/L, magnesium 1.2 mmol/L. Prothrombin time was 15 seconds, and international normalized ratio (INR) was 1.17. Urine toxicology screen was positive for benzodiazepines. Troponin level was 2.2 ng/mL (normal<0.4). Initial blood cultures were drawn in the emergency department and later grew *P. aeruginosa* susceptible to amikacin, aztreonam, cefepime, ciprofloxacin, imipenem, piperacillin/tazobactam and tobramycin. Serology was positive for Hepatitis C infection and negative for HIV infection.

On presentation, he was admitted to the intensive care unit with the diagnosis of septic shock. He was started on intravenous vancomycin, tobramycin, micafungin and cefepime.

Three sets of blood cultures grew *C. parapsilosis* susceptible to fluconazole, itraconazole and 5 flucytosine, and *P. aeruginosa* susceptible to amikacin, aztreonam, cefepime, ciprofloxacin, imipenem, piperacillin/tazobactam and tobramycin.

A 2D echocardiogram showed a bioprosthetic aortic valve with a medium, 10 mm, pedunculated, highly mobile vegetation of the aortic valve attached to the posterior aortic annulus. There was no significant aortic valvular regurgitation. There was mild mitral valvular regurgitation.

At day 2 of hospitalization, a transesophageal echocardiogram was done. It showed no aortic abscess but small, 5 mm, mobile vegetation of the aortic valve attached to the non-coronary cusp without significant regurgitation. It also showed large, 10 mm, mobile mitral valve vegetation attached to the anterior leaflet without significant regurgitation.

The hospital course was complicated by acute renal failure and acute heart failure for which the patient was aggressively resuscitated. He was electively intubated and medically stabilized to undergo valve replacement 5 days later. Ethical and logistic considerations were the focus of an extensive discussion among the different treating medical and surgical teams given the active injecting drug use while being treated for a relapse of infective endocarditis. The decision was finally made to proceed with the valve replacement surgery. The patient was kept in hospital for the duration of intravenous antibiotics to ensure adherence to therapy and to prevent use of the intravenous access for illicit drug use.

At day 7 of hospitalization, he underwent aortic and mitral valve replacement. Cultures from the valves also grew *P. aeruginosa* and *C. parapsilosis.* All blood cultures drawn after day 1 were negative.

Tobramycin and cefepime were given for 6 weeks postoperatively. Tobramycin trough levels were measured frequently to ensure therapeutic levels. Micafungin was given for 5 days postoperatively, and replaced by anidulafungin that was given for 26 days. Fluconazole was given for 30 days postoperatively while in hospital and prescribed for 3 months after discharge from the hospital.

Throughout hospitalization, he remained afebrile and euvolemic without symptoms of heart failure. Two weeks after discharge from the hospital, he returned to the infectious disease clinic, being in good health, without history of fever, excess sweating, chest pain or shortness of breath. Follow-up blood cultures were drawn and were negative.

## Discussion

Polymicrobial endocarditis is a growing problem and has been attracting increasing attention over the past decade [3,4]. The etiology in IVDU is changing, comprising *Staphylococcus*, *Pseudomonas* and other pathogenic fungi [5,6]. Most commonly isolated fungi are *C. albicans* (24%). Non *albicans candida* accounts for another 24% of the fungal isolates [6,7]. The difficulty arises in the management of these cases, especially with the growing resistance to the usual combination antibiotics [8].

This is the first time a patient has presented with *P. aeruginosa* and *C. parapsilosis* endocarditis. Optimal treatment options and cure rate remain to be evaluated. We present a man who had successful treatment with a combination of antibiotics and antifungals in addition to surgical treatment.

## Conclusion

Polymicrobial endocarditis with *C. parapsilosis* and *P. aeruginosa* in intravenous drug users can be cured with surgical valve replacement and extended duration intravenous antibiotics and antifungals.

## Abbreviations

BUN: blood urea nitrogen; IDU: injecting drug user; INR: international normalized ratio; IVDU: intravenous drug use; PIC: peripherally inserted catheter.

## Consent

Written informed consent was obtained from the patient for publication of this case report and any accompanying images. A copy of the written consent is available for review by the Editor-in-Chief of this journal.

## Competing interests

The authors declare that they have no competing interests.

## Authors' contributions

HD initiated the case report, obtained the consent, and gathered the patient's data. FA reviewed the article, provided some data and contributed to the discussion. MZ supervised and edited the case report and provided some of the references.
